# High-Performance Thin Film Composite Nanofiltration (NF) Membrane Constructed on Modified Polyvinylidene Fluoride (PVDF) Substrate

**DOI:** 10.3390/membranes15070216

**Published:** 2025-07-20

**Authors:** Junliang Dong, Qianzhi Sun, Xiaolin Feng, Ruijun Zhang

**Affiliations:** School of Civil and Transportation Engineering, Hebei University of Technology, Tianjin 300401, China

**Keywords:** nanofiltration membrane, substrate, PVDF, hydrophilicity, drinking water treatment

## Abstract

The inherent hydrophobic nature of PVDF material renders it challenging to establish a stable aqueous hydration layer, thereby limiting its suitability as a substrate for the preparation of nanofiltration (NF) membranes. In this study, we developed a novel modification approach that effectively enhances the hydrophilicity of PVDF substrates through the incorporation of sulfonic acid-doped polyaniline (SPANI) and hyperbranched polyester (HPE) into the PVDF casting solution, followed by cross-linking with trimesoyl chloride (TMC). The introduction of SPANI and HPE, which contain reactive polar amino and hydroxyl groups, improved the hydrophilicity of the substrate, while the subsequent cross-linking with TMC effectively anchored these components within the substrate through the covalent linking between TMC and the reactive sites. Additionally, the hydrolysis of TMC yielded non-reactive carboxyl groups, which further enhanced the hydrophilicity of the substrate. As a result, the modified PVDF substrate exhibited improved hydrophilicity, facilitating the construction of an intact polyamide layer. In addition, the fabricated TFC NF membrane demonstrated excellent performance in the advanced treatment of tap water, achieving a total dissolved solid removal rate of 57.9% and a total organic carbon removal rate of 85.3%. This work provides a facile and effective route to modify PVDF substrates for NF membrane fabrication.

## 1. Introduction

As a typical pressure-driven membrane technology ranging between RO and ultrafiltration (UF), nanofiltration (NF) has emerged as a promising method for effectively purifying drinking water, profiting from its simplicity, energy efficiency, affordability, and environmental friendliness [1,2,3]. Compared to porous UF membranes and non-porous RO membranes, NF membranes’ relatively dense selective layer and charged polymer matrix endow them with selectivity for contaminant removal; heavy metals, antibiotics, and other harmful organic substances can be effectively removed while retaining certain minerals that are beneficial to human health [4,5,6,7]. As the cornerstone of NF water purification technology, NF membranes significantly influence both purification efficiency and operational costs [8]. Consequently, many researchers are dedicated to the design and modification of high-performance NF membranes from various perspectives.

Currently, thin film composite (TFC) NF membranes, which feature an ultrathin selective layer formed through interfacial polymerization (IP) on a porous substrate, continue to dominate research interest and industrial applications [9,10]. During the IP process, the substrate is first impregnated with an aqueous solution containing an amine monomer such as piperazine (PIP) or m-phenylenediamine (MPD) [11]. It is then brought into contact with an immiscible organic phase containing acyl chloride (usually trimesoyl chloride, TMC), leading to the formation of a cross-linked polyamide (PA) layer. Most of the studies aimed at enhancing membrane performance primarily concentrated on the regulation of the PA selective layer. A variety of strategies have been explored including the screening of novel monomers [12], the optimization of IP reaction parameters [13], blending with nanomaterials [14], and post-treatment techniques [15]. However, a major obstacle that limits the practical application of these modification strategies is their negative impact on the integrity of the resulting selective layer [16]. In recent years, the design and modification of substrates have emerged as another promising strategy for enhancing the performance of TFC NF membranes [17,18]. However, most of the porous substrates in both reported and commercial TFC NF membranes are composed of polysulfone (PSF) and polyethersulfone (PES), rather than other polymers [19,20,21]. This is primarily due to the challenge of finding cost-effective alternatives that possess the necessary mechanical strength, hydrophilicity, and chemical stability required for the synthesis of TFC NF membranes.

Polyvinylidene fluoride (PVDF) has been widely utilized in the construction of microfiltration (MF) and UF membranes because of its strong thermal stability, mechanical strength, and chemical resistance [22]. However, to the best of our knowledge, no commercially viable TFC NF membranes currently utilize PVDF substrates—a gap attributed to their inherent hydrophobicity. This property critically precludes the formation of a stable aqueous hydration layer, which is essential for synthesizing a defect-free PA selective layer. To address this challenge, several pioneering researchers have proposed various strategies which can be classified into two routes. The first one involves innovating the IP procedure while maintaining the use of a hydrophobic PVDF substrate. For instance, ethanol/acetone can be mixed into the aqueous phase to increase the wettability of PVDF [23,24,25], but the permselectivity of the resultant NF membranes is not desirable. Taking the optimized NF sample prepared by Tang et al. as an example, its water permeance was only 8.1 L m^−2^ h^−1^ bar^−1^ [24]. Different from this idea, Lee at al. proposed to wet the PVDF substrate via the pressurization of the aqueous phase or flowing the organic solution at a specific velocity [26,27]. The second route involves directly regulating the physicochemical properties of the PVDF substrate. Based on Jurin’s law, which states that the pressure exerted by the membrane surface to push water away is inversely proportional to the radius of the membrane pore, a hydrophobic PVDF membrane with a large pore size is expected to become wet by the aqueous phase. Accordingly, Wu et al. successfully prepared a TFC NF membrane on a PVDF substrate with a large pore size up to 100 nm [28]. The resultant NF membrane presented a high water permeance up to 27.0 L m^−2^ h^−1^ bar^−1^, but its Na_2_SO_4_ rejection was only 96.2%. In addition, low-temperature plasmas were employed by Kim et al. to modify a commercial PVDF MF membrane, thus improving its hydrophilicity [29]. However, the oxidation of plasmas might decrease the mechanical strength of the PVDF membrane.

By comparing the two technical routes mentioned above, it is evident that modifying the characteristics of the PVDF substrate is more convenient and feasible, allowing for better compatibility with the existing industrial production processes of TFC NF membranes. To shorten the modification process, hydrophilic fragments can be directly introduced into the PVDF polymer matrix during the non-solvent-induced phase separation (NIPS) process. However, additional measures must be implemented to ensure the stable incorporation of these hydrophilic fragments into the resulting PVDF substrate. Only through such regulatory measures can we obtain a high-performance PVDF-supported TFC NF membrane.

To achieve this vision, in this work, sulfonic acid-doped polyaniline (SPANI) and hyperbranched polyester (HPE), which possess numerous hydrophilic groups and reactive sites, were blended into a PVDF casting solution and subsequently cross-linked with TMC. Subsequently, an intact PA layer can be formed on the modified PVDF substrate through a conventional IP strategy between PIP and TMC. This process can result in the synthesis of a high-performance PVDF-supported TFC NF membrane, which can be used to purify drinking water by further reducing dissolved organic and inorganic contaminants that conventional water treatment technologies cannot remove. Compared to previously reported studies, our strategy is both facile and effective, making it easily adoptable by existing TFC membrane manufacturers.

## 2. Materials and Methods

### 2.1. Materials

The polyvinylidene fluoride (PVDF, Solef^®^ 6020, with a density of 1750~1800 mg/cm^3^) used for membrane preparation was acquired from Solvay. The PET nonwoven fabric used as a support for PVDF membrane formation was obtained from Suka New Material Technology Co., Ltd (Shanghai, China). Hyperbranched polyester (HPE, H30), featuring a molecular weight of 3387.4 g/mol, was generously supplied by Weihai Chenyuan Dendrimer Technology Co., Ltd. (Weihai, China). Sulfonic acid-doped polyaniline (SPANI, 98%) was purchased from Shanghai Maclin Biochemical Technology Co., Ltd. (Shanghai, China). N-methylpyrrolidone (NMP, 99%), which served as the solvent for preparing the casting solution, was also purchased from Shanghai Maclin Biochemical Technology Co., Ltd. Trimesoyl chloride (TMC, >98.0%) was obtained from Tokyo Chemical Industry Co., Ltd. (Shanghai, China). Piperazine (PIP, >99.0%), n-hexane, sodium sulfate (Na_2_SO_4_, >99.0%), sodium chloride (NaCl, >99.0%), and Bovine Serum Albumin (BSA) were all sourced from Aladdin Industrial Corporation Co., Ltd. (Shanghai, China). Unless otherwise specified, all solutions were prepared using ultrapure water produced by a reverse osmosis and ion exchange purification system (RUPF-30, Xinrui, China).

### 2.2. Preparation of Modified PVDF Substrate and PVDF-Supported TFC NF Membrane

PVDF powder, NMP, HPE, and SPANI were used to prepare the casting solution. The PVDF powder intended for membrane preparation should be vacuum-dried for 24 h at a pressure of −0.08 MPa and a temperature of 60 °C. During the preparation of the casting solution, HPE (4 wt%) or SPANI (0.3 wt%) was first added to the NMP solvent, followed by stirring with a magnetic stirrer for 15 min and ultrasound dispersion for 30 min. Afterward, PVDF powder (19 wt%) was added to the mixture and mechanically mixed for 24 h to obtain a homogeneous casting solution. The casting solution should be placed in a vacuum oven, with the pressure set between −0.08 MPa and −0.06 MPa, and then degassed at 25 °C for 24 h to achieve a uniform and stable casting liquid. Then it can be cast onto the PET nonwoven fabric fixed on the glass sheet using a casting knife. The thickness of the casting solution was fixed at 200 μm. The membrane obtained after casting was subsequently immersed in a coagulation bath (pure water at 25 °C) for 24 h to facilitate phase inversion.

For the cross-linking modification of the HPE/SPANI doping PVDF membrane, two strategies were proposed. The first approach involved directly adding TMC (0.2 wt%) into the casting solution prior to phase inversion. The resultant casting solution containing PVDF/NMP/HPE/SPANI/TMC was used to prepare the modified PVDF membrane. This modified membrane was denoted as the HCDM-PVDF substrate. The second approach entailed cross-linking after phase inversion. For this approach, the casting solution containing PVDF/NMP/HPE/SPANI was used to prepare the modified PVDF membrane. After phase inversion, the resultant membrane was further soaked in a TMC hexane solution (0.2 wt%) for 1 min. Then the modified PVDF membrane was denoted as the HCAM-PVDF substrate.

**Figure 1:**
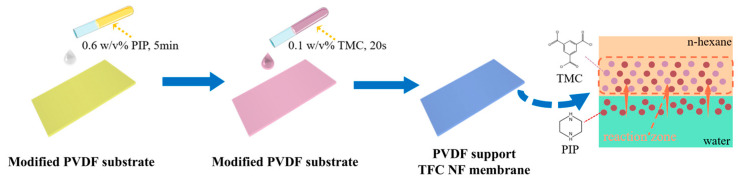
An illustrative diagram of the NF membrane preparation procedure on the modified PVDF substrate.

As shown in Figure 1, the TFC NF membrane was fabricated using the conventional IP method on the HCDM-PVDF or HCAM-PVDF substrate. The aqueous phase solution was prepared by dissolving 0.6 *w*/*v*% PIP in pure water, while the organic phase solution was composed of 0.1 *w*/*v*% TMC dissolved in *n*-hexane. The substrate membrane was first coated with the PIP solution for 5 min, after which excess aqueous solution was removed using a rubber scraper. Next, the TMC *n*-hexane solution was gently poured onto the surface of the substrate to initiate the IP reaction for 20 s, followed by rinsing to remove the residual TMC with pure n-hexane. Finally, the membrane sample was heated in an oven at 50 °C for 3 min to stabilize the membrane structure. The resulting TFC NF membrane was stored in ultrapure water at 4 °C until testing.

### 2.3. Membrane Characterization

The selected membrane sample for characterization was naturally dried in a well-ventilated environment for 24 h, followed by further drying in a vacuum oven at 30 °C and a pressure of −0.06 MPa for an additional 24 h to remove residual moisture. The membrane’s surface and cross-sectional morphology were characterized using a scanning electron microscope (SEM, TESCAN MIRA LMS, Brno, Czech Republic). The hydrophilicity of the membrane surface was assessed by measuring the water contact angle (WCA) using a drop shape analyzer (Dataphysics OCAI5EC, Stuttgart, Germany). The reported WCA value represents the average of five measurements taken at different locations on each membrane sample, with a water droplet volume of 0.2 µL used for the WCA assessment.

### 2.4. Evaluation of Membrane Separation Performance

**Figure 2:**
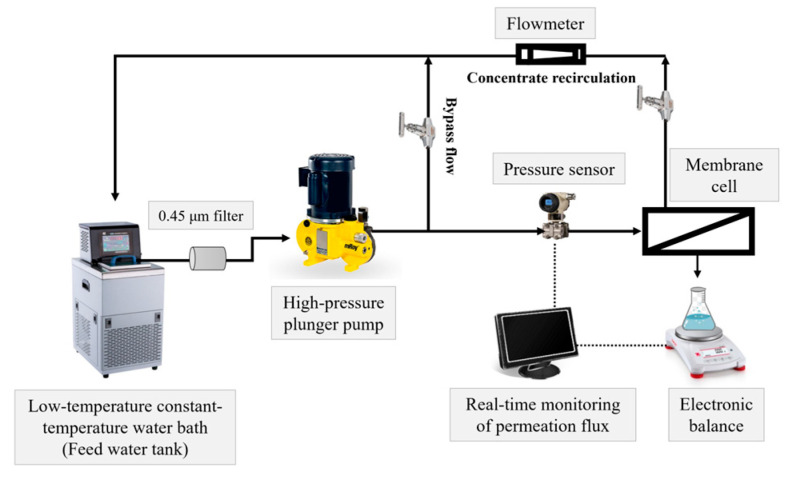
A diagram of the self-made cross-flow membrane filtration system.

Membrane filtration experiments were conducted using a self-made cross-flow membrane filtration system (as illustrated in Figure 2), with the membrane cell sourced from Sterlitech Corporation (CF016P, Auburn, WA, USA). The effective membrane area in this cell was 20.6 cm^2^. For the modified PVDF substrate, the filtration test commenced by pre-pressurizing the membrane with pure water at 2 bar until a steady flux was achieved. Measurements were then conducted under a pressure of 1 bar at 25 °C. In the case of the PVDF-supported TFC NF membrane, the filtration test began with pre-pressurization using pure water at 6 bar until a steady flux was attained. Subsequently, measurements were performed at 5 bar and 25 °C, with a cross-flow velocity of 0.45 m/s to reduce concentration polarization. The pure water permeance (PWP, L·m^−2^·h^−1^·bar^−1^) was calculated according to Equation (1). In this equation, A is the effective membrane area (m^2^), Δt is the time interval (h), ΔP is the trans-membrane pressure (bar), and V is the permeate volume (L).

(1) $$PWP=\frac{V}{A\times ∆t\times ∆P}$$

BSA is a large (~66.5 kDa), monomeric, globular protein which is highly soluble in water and buffers over a wide pH range. It has a hydrodynamic diameter of approximately 7 nm and a globular structure, making it an ideal molecular probe for evaluating pore sizes in ultrafiltration (UF) membranes. This is particularly relevant for UF membranes with a molecular weight cut-off (MWCO) of 50~100 kDa, as their pore sizes align well with BSA’s dimensions. Notably, UF membranes in this MWCO range are optimal substrates for TFC NF membrane preparation. Therefore, in this work, 200 mg/L BSA was used to assess the selectivity of modified PVDF substrates. Subsequently, 2000 mg/L Na_2_SO_4_ was employed to evaluate the salt rejection of the PVDF-supported TFC NF membrane. The observed solute rejection (R, %) was calculated according to Equation (2). The BSA concentration was determined using ultraviolet spectrophotometry, while the Na_2_SO_4_ concentration was assessed by measuring the conductivities of both the feed and permeate solutions with a conductivity meter (Ohaus ST3100C/F, Pine Brook, NJ, USA). In the following equation, Cf is the solute concentration in feed, and Cp is the solute concentration in the permeate.

(2) $$R=\left(1-\frac{C_{p}}{C_{f}}\right)\times 100\%$$

The PVDF-supported TFC NF membrane was further utilized to treat tap water sourced from the municipal network in the Hongqiao District of Tianjin, China. The main quality indicators of this tap water are presented in Table 1. The pH of the water sample was measured using a calibrated pH meter (Lei-ci, DZS-706A, Shanghai, China), while the concentrations of various ions were analyzed using ion chromatography (Metrohm 883 Basic IC plus, Herisau, Switzerland). The total organic carbon (TOC) content was determined using a TOC analyzer (TOC-L CPH, Shimadzu, Kyoto, Japan). It can guarantee a 100% oxidation recovery rate for all carbon in the sample through catalytic combustion at a fixed temperature of 680 °C. A high-sensitivity NDIR (non-dispersive infrared detector), combined with a large sample volume combustion system, enables the detection of TOC at levels as low as 10 µg/L.

## 3. Results and Discussion

### 3.1. Influence of SPANI/HPE Doping Modification on PVDF Substrate

**Figure 3:**
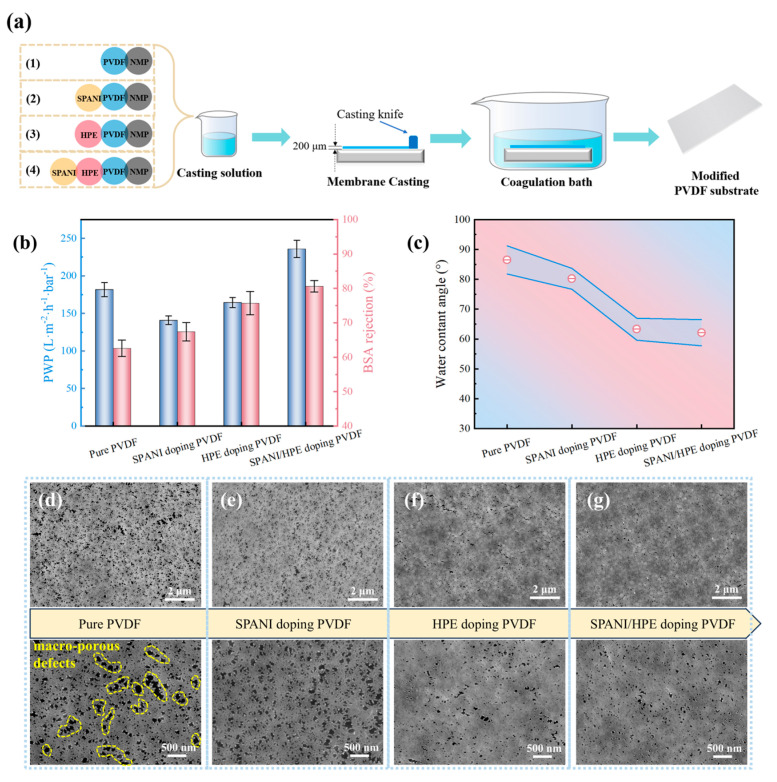
Property evaluation of PVDF-based substrate. (**a**) Illustrative diagram of preparation procedure. (**b**) Separation performance (**c**) water contact angle and (**d**–**g**) SEM surface morphology of modified PVDF substrates.

To identify the influence of SPANI/HPE doping modification on the PVDF substrate, four kinds of substrates were prepared, as illustrated in Figure 3a. Then the separation performance and physicochemical properties of the resultant membranes were characterized. As shown in Figure 3b, the pure PVDF substrate without any dopant exhibited a PWP around 185 L m^−2^ h^−1^ bar^−1^ and a poor BSA rejection of ~62%. This inferior separation performance can be attributed to the presence of macro-porous defects observed in the SEM image of Figure 3d, which facilitates the passage of water as well as BSA molecules. Additionally, a remarkable high water contact angle (WCA) of nearly 90° can be observed in Figure 3c for the pure PVDF substrate, indicating the inherent hydrophobicity of PVDF material.

After the addition of SPANI into the PVDF casting solution, the membrane’s PWP decreased, while its BSA rejection level increased. However, the changes in surface WCA (Figure 3c) and SEM morphology (Figure 3e) were not significant. This suggests that the doping modification with SPANI did not affect the surface properties of the membrane. The observed changes in separation performance are likely due to the accumulation of SPANI particles within the inner pore structures of PVDF. Unlike SPANI, HPE doping could simultaneously alter both the separation performance and surface characteristics of the membrane. As shown in Figure 3b, the BSA rejection of HPE doping PVDF membranes obviously improved, accompanied by a slight decrease in PWP. Meanwhile, the water contact angle sharply decreased to 63.3°. In addition, as shown in Figure 3f, the large pore defects were eliminated, and membrane surface porosity was reduced.

To harness the modification effects of both SPANI and HPE, a substrate denoted as SPANI/HPE doping PVDF was prepared and tested. Interestingly, there seems to be some synergistic modification effect between SPANI and HPE. In comparison to HPE doping PVDF, SPANI/HPE doping PVDF presented a similar water contact angle and more even pore size distribution. Consequently, a significant enhancement in both PWP (~230 L m^−2^ h^−1^ bar^−1^) and BSA rejection (~81%) was produced, as shown in Figure 3b. However, the BSA rejection rate of 81% was still inadequate, and the water contact angle of 62.1° was not sufficiently low. According to previous research [30,31,32], the water contact angle of a substrate for TFC NF membrane preparation should be lower than 50°.

### 3.2. Effect of TMC Cross-Linking on Modification of PVDF Substrate

**Figure 4:**
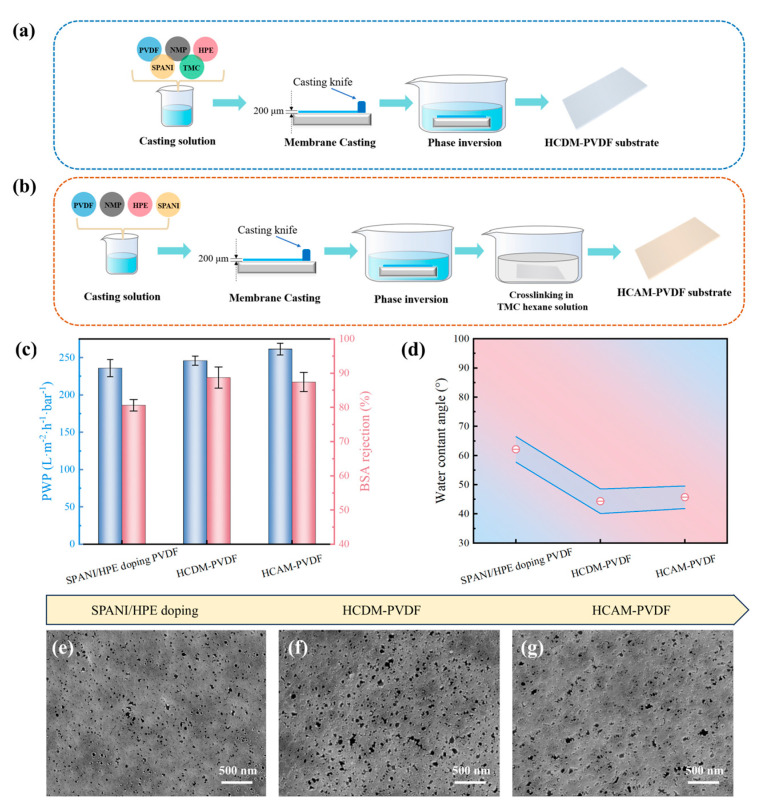
Property evaluation of TMC cross-linking PVDF substrates. Illustrative diagram of preparation procedure of (**a**) HCDM-PVDF substrate and (**b**) HVAM-PVDF substrate. (**c**) Separation performance, (**d**) water contact angle, and (**e**–**g**) SEM surface morphology of TMC cross-linking PVDF substrates.

To further optimize the properties of the PVDF substrate, two types of TMC cross-linking methods were employed for subsequent modifications. As depicted in Figure 4a,b, the first method involves directly incorporating TMC into the PVDF casting solution prior to phase inversion, referred to as HCDM-PVDF. The second method entails immersing the PVDF substrate into the TMC/hexane solution after phase inversion, termed HCAM-PVDF.

The separation performance and physicochemical properties of the TMC-modified PVDF substrate were characterized. As presented in Figure 4c, compared to the uncross-linked PVDF substrate, both the HCDM-PVDF and HCAM-PVDF substrates exhibited slightly enhanced PWP and BSA rejection. Meanwhile, Figure 4d demonstrates a substantial decline in WCA (~45°) for the TMC cross-linked substrate. This decline can be attributed to the susceptibility of the acyl chloride functional groups in the TMC molecule to hydrolysis and their conversion to carboxyl groups, which substantially increases the hydrophilicity of the PVDF substrate. In addition, SEM images (Figure 4e–g) revealed a notable increase in pore structures on the substrate following TMC modification. Notably, the BSA rejection level (~88%) of the HCDM-PVDF substrate and its hydrophilicity (44.3°) were slightly greater than those of the HCAM-PVDF substrate (~87%, 45.7°). Additionally, The SEM morphology of the HCDM-PVDF substrate revealed a higher density of pore structures compared to the HCAM-PVDF substrate. These findings indicate that the direct incorporation of TMC into the PVDF casting solution for the HCDM-PVDF substrate could bring a better modification effect. Moreover, the first cross-linking modification strategy, which involves directly adding TMC to the PVDF casting solution before phase inversion, is more convenient and cost-effective.

### 3.3. Impact of PVDF Substrate on Performance of Resultant TFC NF Membranes

**Figure 5:**
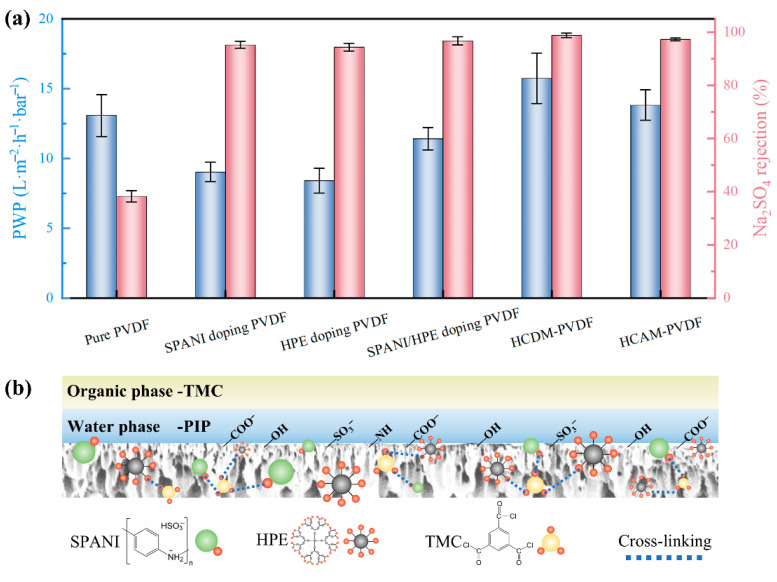
(**a**) Separation performance of fabricated NF membrane. (**b**) Illustrative diagram of impact of substrate on resultant TFC NF membrane.

The preceding sections confirmed the effectiveness of SPANI/HPE doping followed by TMC modification in enhancing the hydrophilicity and optimizing the pore structure of PVDF substrates. To assess the impact of these modification processes on interfacial polymerization, six types of PVDF-based substrates were utilized to prepare the TFC NF membrane. The separation performance of six resultant NF membranes is shown in Figure 5a. The pure PVDF substrate, due to its inherent hydrophobicity, yielded an NF membrane with a low PWP of 13.1 L m^−2^ h^−1^ bar^−1^ and an inferior Na_2_SO_4_ rejection of only 38.2%. Doping SPANI or HPE into the PVDF substrate led to a typical “trade-off” effect, where a significant enhancement in the Na_2_SO_4_ rejection (~95%) of the fabricated NF membrane was achieved at the expense of reduced PWP (8 L m^−2^ h^−1^ bar^−1^). Interestingly, the alliance of SPANI and HPE further slightly increased the PWP of the fabricated NF membrane, likely due to the higher PWP of the SPANI/HPE doping PVDF substrate. Notably, the cross-linking modification step involving TMC breaks through the “trade-off” limitation, wherein both PWP and Na_2_SO_4_ rejection were concurrently enhanced. During the two TMC modification methods, the HCDM-PVDF substrate, where TMC was directly incorporated into the casting solution, demonstrated better NF performance based on its enhanced modification effect. Specifically, the NF membrane fabricated with the HCDM-PVDF substrate exhibited a high PWP of 15.7 L m^−2^ h^−1^ bar^−1^ alongside a remarkable Na_2_SO_4_ rejection up to 98.8%. By comparison, the PVDF-supported TFC NF membrane reported by Tang et al. exhibited a lower PWP of 8.1 L m^−2^ h^−1^ bar^−1^ [24]. Similarly, while Wu et al. achieved a higher PWP of 27.0 L m^−2^ h^−1^ bar^−1^ using a PVDF substrate with larger pore size, their Na_2_SO_4_ rejection was limited to 96.2% [28]. Therefore, it can be concluded that our modification strategy achieved a good balance between water permeability and selectivity.

As the TFC NF membrane constructed on the HCDM-PVDF substrate presented the best separation performance, its modification mechanism was highlighted and is illustrated in Figure 5b to elucidate the impact of the substrate on NF membrane formation. The typical NF membrane formation process involves the sequential immersion of the substrate in aqueous PIP and organic TMC solutions, driving interfacial polymerization (IP) between PIP and TMC on the stable hydration layer of the substrate to form an intact selective layer. In this study, the substrate was modified with SPANI, HPE, and TMC to enhance its properties. The introduction of SPANI and HPE, which contain reactive polar amino and hydroxyl groups, respectively, enhanced the hydrophilicity of the substrate and created additional reactive sites for TMC coordination, as the functional groups of SPANI and HPE readily react with the acyl chlorine groups of TMC. Additionally, the PVDF substrate was cross-linked with TMC, which covalently bonded with the reactive sites of SPANI and HPE, effectively anchoring these components within the substrate. In addition, TMC underwent hydrolysis, yielding non-reactive carboxyl groups that further enhanced the substrate’s hydrophilicity. Consequently, the improved hydrophilicity of the PVDF substrate facilitated the formation of a stable hydration layer on its surface, which in turn contributed to the creation of an intact selective layer. Moreover, the reactive sites introduced by SPANI and HPE promoted additional reactions that replenished defects, thereby promoting the formation of a high-performance NF membrane.

### 3.4. Performance Evaluation of PVDF-Supported TFC NF Membrane

**Figure 6:**
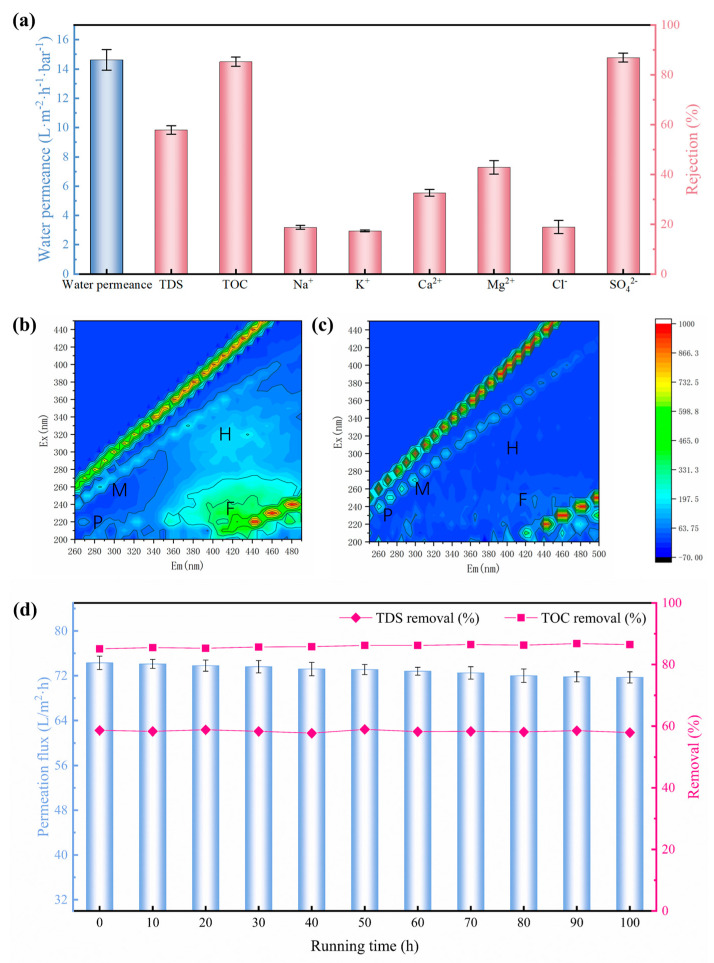
(**a**) Tap water treatment performance. (**b**) Tap water and (**c**) NF membrane effluent measured by three-dimensional fluorescence. (**d**) Long-term tap water filtration performance of PVDF-supported TFC NF membrane tested at 5 bar and 25 °C.

The demand for high-quality drinking water has catalyzed the focus on advanced treatment technologies for municipal tap water production [33]. Taking the group Health Drinking Water Quality Standard (T/BJWA 001-2021) [34] in China as example, it imposes more stringent requirements, specifying TDS levels of 50~300 mg/L and TOC ≤ 1.0 mg/L. In the context of this trend, NF technology has emerged as a promising candidate due to its high-quality effluent [35]. To assess the viability of the NF membrane fabricated on the HCDM-PVDF substrate, it was used to treat the tap water sourced from the municipal network in the Hongqiao District of Tianjin, China. The main quality indicators of this tap water are presented in Table 1. As depicted in Figure 6a, this NF membrane exhibited a notable tap water permeance of 15 L m^−2^ h^−1^ bar^−1^, alongside a moderate total dissolved solid (TDS) removal rate of 57.9%. Notably, the rejection rates of bivalent ions (SO_4_^2−^ (86.9%), Mg^2+^ (42.9%), and Ca^2+^ (32.6%)) were significantly higher than those of monovalent ions (Na^+^ (18.7%), K^+^ (17.3%), and Cl^−^ (18.9%)). This discrepancy can be attributed to the larger hydration diameter and electric charge of the bivalent ions. Additionally, the organic matter removal efficiency of the NF membrane was assessed through TOC rejection, which revealed an excellent removal effect for TOC up to 85.3%. As shown in Table 1, the TOC of raw tap water was approximately 2.2 mg/L. After the NF treatment, this level was reduced to 0.32 mg/L, effectively meeting the high-quality drinking water standard of TOC < 1 mg/L. This proficiency in organic matter removal is conducive to ensuring the safety of drinking water. In addition, Excitation Emission Matrix (EEM) Spectra were employed to quantify the organic matter present within tap water before and after the NF treatment. In general, the fluorescence response regions of the EEM can be categorized as M, P, H, and F, corresponding to microbial products, proteins, humic acids, and fulvic acids, respectively [36]. As demonstrated in Figure 6b, only small amounts of fulvic acid (F region) and humic acid (H region) were detected in the raw tap water, reflecting its relatively clean quality. It is well known that fulvic acid and humic acid are types of natural organic matter (NOM). Despite their diversity and complexity, they typically possess large molecular weights. Therefore, the NF membrane is expected to effectively remove these substances with >96% rejection [37,38]. Consequently, as shown in Figure 6c, these fractions were detected at nearly negligible levels in the NF permeate water. These results demonstrated that the NF membrane constructed on the HCDM-PVDF substrate can significantly reduce the content of organic impurities in tap water, underscoring its exceptional ability to produce high-quality drinking water.

To evaluate the long-term stability of the PVDF-supported TFC NF membrane, we conducted a continuous 100 h tap water filtration experiment at 5 bar and 25 °C. Throughout this extended operation, water permeation flux, TDS removal, and TOC removal were monitored at 10 h intervals. The results in Figure 6d demonstrate exceptional operational stability: permeation flux decreased marginally from 74.3 to 71.7 L·m^−2^·h^−1^, while TDS and TOC rejection remained consistently high at approximately 58.3% and 85.9%, respectively. This robust performance confirms the system’s long-term stability potential.

## 4. Conclusions

In this study, we successfully enhanced the hydrophilicity of the PVDF substrate by incorporating SPANI and HPE into the casting solution, followed by cross-linking with TMC. This modification rendered the substrate highly suitable for constructing high-performance TFC NF membranes. The presence of reactive polar groups (amino from SPANI and hydroxyl from HPE) significantly decreased the WCA from 86.5° to 62.1°. The subsequent TMC cross-linking effectively anchored these hydrophilic components within the substrate matrix and introduced non-reactive carboxyl groups via hydrolysis, further reducing the WCA to 44.3°. Crucially, this enhanced hydrophilicity promoted the formation of a dense and intact PA selective layer. The resulting TFC NF membrane exhibited desirable performance in advanced tap water treatment, achieving proper removal rates of 57.9% for TDS and 85.3% for TOC. This enables the permeate (TDS: ~65.3 mg/L; TOC: ~0.32 mg/L) to meet the stringent requirements of the Chinese group Health Drinking Water Quality Standard (T/BJWA 001-2021). Beyond offering a straightforward and efficient method for PVDF substrate hydrophilization, the proposed approach in this work could be adapted to modify other hydrophobic polymer substrates in future studies. Additionally, broader feed water testing, such as wastewater containing specific organic pollutants and brackish water with higher ionic strength, is also necessary to validate its robustness and to identify suitable application niches.

## Figures and Tables

**Table 1:** Water quality indicators of tap water.

Index	Content	Unit
pH	7.4~7.7	-
TOC	2.2	mg/L
TDS	155.2	mg/L
Na^+^	9.32	mg/L
K^+^	2.19	mg/L
Ca^2+^	45.33	mg/L
Mg^2+^	7.45	mg/L
Cl^−^	8.99	mg/L
SO_4_^2−^	29.34	mg/L

## Data Availability

Data is unavailable due to privacy or ethical restrictions.
